# Obesity and COVID-19: Oro-Naso-Sensory Perception

**DOI:** 10.3390/jcm9072158

**Published:** 2020-07-08

**Authors:** Amira Sayed Khan, Aziz Hichami, Naim Akhtar Khan

**Affiliations:** Centre de Recherche Inserm, U1231 INSERM/UB/AgroSup, Team—Physiologie de la Nutrition & Toxicologie, Université de Bourgogne Franche-Comté (UBFC), Faculté des Sciences de la Vie, 6 Boulevard Gabriel, 21000 Dijon, France; amira.khan@u-bourgogne.fr (A.S.K.); aziz.hichami@u-bourgogne.fr (A.H.)

**Keywords:** taste, smell, COVID-19, SARS-CoV-2, obesity

## Abstract

Through a recent upsurge of severe acute respiratory syndrome coronavirus-2 (SARS-CoV-2) pandemic, the clinical assessment of most of the coronavirus disease 19 (COVID-19) patients clearly presents a health condition with the loss of oro-naso-sensory (ONS) perception, responsible for the detection of flavor and savor. These changes include anosmia and dysgeusia. In some cases, these clinical manifestations appear even before the general flu-like symptoms, e.g., sore throat, thoracic oppression and fever. There is no direct report available on the loss of these chemical senses in obese COVID-19 patients. Interestingly, obesity has been shown to be associated with low ONS cues. These alterations in obese subjects are due to obesity-induced altered expression of olfacto-taste receptors. Besides, obesity may further aggravate the SARS-CoV-2 infection, as this pathology is associated with a high degree of inflammation/immunosuppression and reduced protection against viral infections. Hence, obesity represents a great risk factor for SARS-CoV-2 infection, as it may hide the viral-associated altered ONS symptoms, thus leading to a high mortality rate in these subjects.

## 1. Introduction

In the month of December 2019, there was an uprising of pneumonia, marked with respiratory distress, among the residents of Wuhan district, located in the north-east of China [1]. The virus responsible for this health disaster was identified as severe acute respiratory syndrome coronavirus-2 (SARS-CoV-2) which belonged to the single-stranded enveloped RNA viruses, and the disease was termed as coronavirus disease 2019 (COVID-19) [2]. It is surprising that in the beginning of the pandemic, most of the COVID-19 patients in Wuhan (China) had some primary health problems, including obesity [1].

## 2. Obesity and Reduced Viral Protection

A recent cohort, conducted in 12 hospitals of the New York state on COVID-19 patients, has proposed that there were 41% obese patients, admitted between March 1, 2020 and April 4, 2020 [3]. The incidence of obesity is increasing steadily in all the corners of the world, with 650 million clinically ill subjects requiring either a surgical or medical treatment [4]. The management of obesity has become a challenging task because this pathology is a favorable ground for several chronic diseases, including cardiovascular complications, type-2 diabetes mellitus, cancer, atherosclerosis, arthrosis and renal dysfunction, and respiratory tract infections (RTI) in virus-affected patients [5,6,7]. The RTI are the main physiological targets in COVID-19 illness [1]. We would like to recall that during 2009 influenza pandemic, obesity was associated with reduced pulmonary immune defenses against the virus [8]. Indeed, obese subjects were not only more prone to infection with the influenza (H1N1) virus, but also developed post-infection severity of illness [9]. An increase in adiposity has been shown to alter the integrity of respiratory epithelium, which might lead to dysfunctional airway fluxes [10]. Due to high weight load with excessive pressure on belly and thorax, obesity will contribute to reduced pulmonary gas exchange capacities, such as forced expiratory volume (FEV) and forced vital capacity (FVC). The experiments conducted on mice have suggested that obesity is associated with high lung permeability [11]. Epidemiological data confirm that there is an increased rate of pneumonia and RTI in COVID-19 obese patients [12]. In fact, the first report on RTI in obese subjects was published by a French team wherein 47% of COVID-19 patients were found to be obese with a high degree (nearly 90%) of artificial ventilation [13].

**Figure 1:**
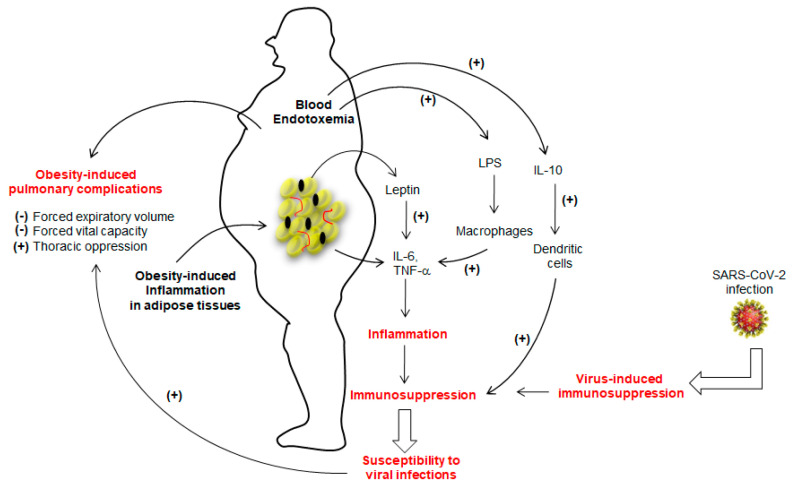
The figure shows the immunosuppression in obese subjects. The adipose tissue of the obese is highly inflamed and, consequently, releases a number of cytokines, particularly IL-6 and TNF-α. whose secretion is further potentiated by leptin. The lipopolysaccharide (LPS)-triggered endotoxemia further aggravates inflammatory condition by inducing the release of IL-6 and TNF-α from macrophages via TLR4 activation. Obesity is also marked with high production of IL-10, which decreases the function of dendritic cells. The prolonged inflammation will lead to immunosuppression that may favor the viral infection. Severe acute respiratory syndrome coronavirus-2 (SARS-CoV-2) has also been shown to induce immunosuppression. Once installed, SARS-CoV-2 will aggravate the obesity-induced lung dysfunctions. (+) and (−) show, respectively, stimulatory and inhibitory actions.

The marked inflammation leading to immunosuppression in obesity seems to favor viral infections [14,15,16]. Sheridan et al. [17] observed that high body mass index (BMI) was associated with a high decline in influenza antibody titers and decreased CD8^+^ T-cell activation after 12 months post-vaccination. As far as SARS-CoV-2 infection is concerned, Tan et al. [18] assessed immunological alterations in COVID-19 patients, wherein they noted an overall decline in CD4^+^ T-cells, CD8^+^ T-cells, B cells and natural killer (NK) cells. Moreover, the number of immunosuppressive T-regulatory, T-reg (CD4^+^CD25^+^Foxp3^+^) cells and concentrations of IL-6, IL-10, and C-reactive protein (CRP) were up-regulated in patients with severe COVID-19 [18], suggesting that SARS-CoV-2 infection may lead to “over-immunosuppression” in the case of obesity (Figure 1).

Since dendritic cells (DCs) are the key players in the regulation of Th1/Th2 dichotomy and T-cell tolerance, their importance to trigger an anti-viral response has been considered primordial [19]. O’Shea et al. [20] have demonstrated that obesity impacts the functions of these cells to trigger appropriate T-cell responses. This interesting report further showed that not only the number of circulating DCs were significantly lower in obese participants than lean subjects, but also in vitro activated-DCs from obese participants expressed less CD83 (a DCs maturation marker) and also produced, in high quantities, the IL-10, an immunosuppressive cytokine [21]. The IL-10, in turn, has been shown to inhibit the ability of DCs to stimulate CD4^+^ T-cells and to downregulate MHC-II, CD86 (a co-stimulatory signal protein), and antigen presentation to CD4^+^ T-cells [21]. Obesity is also marked with high concentrations of leptin, which is also known to trigger the production of IL-6 and TNF-α from adipose tissues (Figure 1) and to increase the risk for viral infection. Indeed, TNF-α administration in mice favors the induction of an experimental autoimmune disease [22]. The adipose tissue is the main source of circulating TNF-α in obesity, as its synthesis is increased by adipocytes in obese subjects and a weight-loss results in its low concentrations [23]. In obesity, leptin further decreases the secretion of adiponectin, an anti-inflammatory adipokine. In fact, the adipose tissue of obese subjects is an inflammatory “hot spot” that is also infiltrated by macrophages [24]. Besides, obesity is also marked with a change in gut microbiota that leaks the entry of lipopolysaccharide (LPS) into blood circulation. The LPS is directly responsible for endotoxemia, so-called, “low grade inflammation”, via Toll-like receptor-4 (TLR-4), by inducing the production of IL-1β, TNF-α and IL-6 from macrophages and, at the same time, some of the adipocytes are also differentiated into “macrophage-like” cells [25]. Finally, we can state that IL-6 and TNF-α are the main players of inflammation in obesity (Figure 1). These two cytokines, along with IL-1β via the NF-kB pathway, have been proposed to be the major cause of immunosuppression [26] as they induce accumulation and activation of myeloid-derived suppressor cells (MDSCs) whose expansion interrupts the maturation of macrophages, DCs and granulocytes [27].

Obesity is also associated with other immunosuppressive landmarks, such as low lymphocyte subset counts and their decreased polyclonal proliferation and oxidative burst activity of monocytes, increased thymic aging, and reduced T-cell repertoire diversity, which lead to increased risk for viral infections and RTI both in experimental models and clinical studies [28]. Luzi and Radaelli [29] have proposed that there would be high viral shedding in obese subjects, thus increasing the probabilities of spreading the viral infection. It is also noteworthy that obesity, complicated by diabetes, may further aggravate the patient’s health status. Indeed, Bello-Chavolla et al. [30] have tried to establish a link between obesity and diabetic condition in SARS-CoV-2 infection. These investigators concluded that obesity might increase the lethality of COVID-19 in diabetic subjects. Diabetes, due to the deleterious role of hyperglycemia on immune responses, represents a risk factor for COVID-19 infection in obesity [31,32]. A French nationwide study, CORONADO (Coronavirus SARS-CoV-2 and Diabetes Outcomes), has clearly shown the deleterious role of obesity in life-threatening outcomes in a large diabetic population with COVID-19 [33,34].

A perusal of above-mentioned studies clearly demonstrates that chronic inflammation, leading to immunosuppression, may contribute to decreased protection against viral infections in obese subjects [35].

## 3. COVID-19 and Reduced Oro-Naso-Sensory (ONS) Perception

It has been recently reported that a significant number of COVID-19 patients suffer from a sudden loss of their senses of smell and taste, even in clinical conditions that are not marked with common viral symptoms such as fever, dry cough or thoracic oppression [36,37].

**Figure 2:**
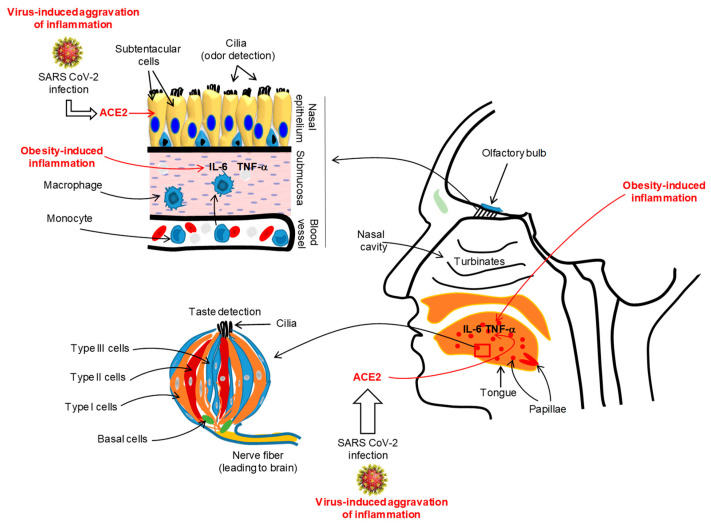
Illustration of the SARS-CoV-2-induced inflammation in oro-naso epithelia. The viral infection via angiotensin converting enzyme 2 (ACE2) may trigger an inflammatory state in the lingual gustatory papillae and olfactory submucosa, affecting, simultaneously, the integrity and functions of taste bud cells and olfactory neurons. The figure shows a taste bud that is constituted of different cell types, such as type I (glial-like), type II (also called, taste receptor cells, TRC), type III (neuron-like) and basal cells (involved in the renewal of all taste bud cell populations). The duration and intensity of SARS-CoV-2-induced inflammation will also depend on pre-existing inflammation (like in obesity) and genetic or epigenetic backgrounds of the subjects. For simplification, we do not show the structure of the tongue papillae. We show a taste bud that is the unit of lingual gustatory papillae. During viral-induced inflammation, the oro-nasal epithelia will be infiltrated by macrophages that will release the pro-inflammatory cytokines such as IL-6 and TNF-α that may aggravate the epithelial integrity and lead to clinical symptoms such as loss of oro-naso-sensory (ONS) functions.

A large number of COVID-19 patients (from 60% to 80%) from Iran have complained of a complete loss of their sense of smell or taste [38]. A multicentric European study conducted on COVID-19 patients demonstrated that nearly 87% of patients reported olfacto-gustatory dysfunctions [39]. A recent meta-analysis on COVID-19 patients, incorporating 10 research articles from 7 countries, has reported that nearly 52% and 43% of them had, respectively, gustatory and olfactory dysfunctions [40]. In France, Gautier and Ravussin [41] reported that there was a sudden appearance of anosmia and/or ageusia in a small number of COVID-19 patients. Similarly, almost two-thirds of COVID-19 patients from Germany also complained of anosmia [42]. In the USA, a survey was performed in the month of April 2020 on COVID-19 patients, and 37.7% of participants complained of altered smell and taste perception [43]. Interestingly, the changes in gusto-olfactory perception in COVID-19 patients were more prevalent in home-quarantined subjects, independently of age and gender [44]. It is important to mention that SARS-CoV-2 does not generate clinically significant nasal congestion or rhinorrhea as seen in general nasal infections [45,46,47,48]. Does SARS-CoV-2 infect taste buds or nasal mucosal epithelia? A recent report, conducted in mice, has demonstrated that mouse sustentacular cells, involved in the transfer of odorant messages to olfactory neurons, express angiotensin converting enzyme 2 (ACE2), which is a port of entry of SARS-CoV-2 (Figure 2) [49].

Beside the implication of ACE2, the viral-induced generalized inflammation in COVID-19 patients would also affect the integrity of the olfactory epithelium. Chronic rhinosinusitis has been shown to trigger alterations in the olfactory mucosa, such as goblet cell hyperplasia, squamous metaplasia, and loss of supporting cells and olfactory neurons, associated with infiltration of pro-inflammatory immune cells [50]. We propose that SARS-CoV-2 might affect the integrity or regeneration/renewal of the olfactory epithelium, impacting the physiological function of olfactory sensory neurons (Figure 2). Hence, we can cite the example of Sendai virus which has been shown to impair olfaction by reducing the regeneration of the olfactory epithelium and olfactory bulb in the mouse [51]. In in vitro experiments on murine olfactory neurons infected with this virus, the number of odorant-responsive cells were decreased. By using a plausible transgenic mouse model, Lane et al. [52] have demonstrated that the induction of TNF-α expression triggered inflammation in the olfactory epithelium and the reversal of TNF-α expression restored the olfactory function in these animals, demonstrating that inflammation is an important factor involved in the loss of olfactory sensory neurons and olfaction sensitivity. The olfactory mucosa is very sensitive to macrophage-secreted inflammatory cytokines, such as macrophage inflammatory protein-1α (MIP-1a) and monocyte chemoattractant protein-1 (MCP-1), that may influence the renewal/regeneration of nasal epithelial cells [53].

As regards taste dysfunction, ACE2 was highly expressed by tongue epithelial cells, but to a lesser extent by buccal and other tissues of the mouth cavity [54]. These observations suggest that the tongue is equipped with a SARS-Cov-2 entry route, but we do not know whether taste papillae and taste bud cells (TBCs) express the ACE2 receptor. We would like to introduce Toll-like receptors (TLRs) that act as receptors for viral RNA, and are abundantly expressed on taste bud cells, particularly on type II and type III cells [55]. The activation of TLRs by the administration of exogenous IFN-γ led to inflammation in taste bud cells and, consequently, to cell death. The autoimmune pathologies in humans or experimental rodent models have clearly demonstrated that inflammation, associated with infiltration by IL-6 and IFN-γ in gustatory epithelium, impacts taste perception [56,57,58]. Moreover, administration of exogenous IFN-γ, via STAT-1 signaling, induced apoptosis of taste bud cells [59]. These observations strongly support that oral taste papillae inflammation may contribute to low oro-sensory perception of sapid molecules.

Beside the peripheral mechanism, different brain areas might be involved in the loss of taste and smell in COVID-19. There are several reports indicating that COVID-19 patients also suffer from neurological complications, such as skeletal muscle injury, delirium and acute cerebrovascular disease [47]. Chigr et al. [60] have proposed that this virus might accede to the olfactory cortex either by the nasopharyngeal cavity or directly by hematogenous spread. There is no direct report on the entry of SARS-CoV-2 into the brainstem; however, clinical features such as vomiting, nausea and loss of appetite suggest a perturbation in the dorsal vagal complex (DVC), which belongs to the medulla oblongata, the lowest region of the brainstem that controls several physiological functions, including food intake. In the DVC, the nucleus of tractus solitaris (NTS) is known to regulate food intake, not only via the vagus nerve that connects the gut, but also via chorda tympani and glossopharyngeal nerves that connect directly to the gustatory taste papillae in the tongue [61]. Ralli et al. [62] have proposed that SARS-CoV-2 could infect the olfactory receptors in the nasal epithelium, through which it may travel to the olfactory bulb and certain brain structures, such as the medulla oblongata. This hypothesis was based on the observations in animal experiments wherein intranasal administration of SARS-CoV, a strain similar to SARS-CoV-2, could enter the brain via the olfactory nerves and spread to the thalamus and brainstem [48]. SARS-CoV-2, in analogy to SARS-CoV34 and MERS-CoV13 infection in transgenic mice, might attain the brainstem [63]. Indeed, using the murine model of HCoV infection, it was shown that SARS and OC43 were able to enter the olfactory bulb via the nasal route and reach the central nervous system (CNS) [64]. Moreover, CT scans and MRI of COVID-19 patients demonstrated “bilateral inflammatory obstruction of the olfactory clefts” [65]. Though we do not have experimental animal data on SARS-CoV-2 entry, we can state that SARS-CoV-2 may enter the CNS, using the olfactory pathway [63], and exert its action via ACE2 that has been detected in the central nervous system [66].

The question arises whether the loss of ONS perception can be considered as an early marker of SARS-CoV-2 infection. We should be very cautious in this regard, as the methods that have been used for the assessment of ONS defects are self-reported examinations. Generally, the investigators employ either a 3-armed forced choice (3-AFC) test or a comparison with 6-*n*-propylthiouracil (PROP) tasting with and without sodium chloride for oral chemosensory perception, and for the detection of olfactory thresholds, rose smell and *n*-butanol are employed. By using these techniques, one can be sure about the decrease (or increase) in taste detection thresholds. However, in none of the reports on COVID-19 patients, such tests were employed. Why do all the COVID-19 subjects not complain of the loss of smell? Is there any genetic or epigenetic predisposition?

## 4. Obesity and Reduced ONS Perception

Before going into detail, we would like to emphasize that a reduced oro-sensory perception would trigger high consumption of palatable food, thus either leading to obesity or worsening this pathology [67,68], though we should not ignore the implication of the food addiction component, particularly for sweet food and those rich in fat [69,70]. The studies conducted on healthy and obese participants suggested that the latter group exhibited lower sensitivity than the former for sweet and sour taste [71]. Diet-induced obesity, by maintaining mice on a high-fat diet for ten weeks, resulted in low taste bud cell number and taste-evoked calcium signaling in obese mice [72]. Similar observations have been reported for bitter and salt tastes in obese subjects [73]. As regards fat taste perception, there was a decreased perception of dietary fatty acids in obese rodents and human beings [61,74,75]. The decrease in taste sensitivity to different taste qualities might be due to partially functional taste receptors/sensors, caused by obesity-induced downregulation [75], genetic polymorphism [75,76,77,78] or epigenetic signatures [79].

The olfaction is not only important for the detection of sense of smell, but also to appreciate the palatability of a hedonic food, as the retro-nasal detection of flavors is brought about by nasal sensory epithelial cells [80,81]. As regards the olfactory cue, there was a significant influence of BMI on olfactory thresholds, which were increased with increasing body weight in obese subjects [82,83]. Patel et al. [84] reported that high BMI was associated with subjective olfactory dysfunction in obese patients. By employing the olfactory threshold-discrimination-identification (TDI) test, Pastor et al. [85] observed that olfactory discrimination power was lesser in obese subjects than control participants. Like taste modalities, the genetic polymorphism of olfactory receptor genes [85,86] or their hypermethylation [87], also contributes to obesity. The decreased smell perception in obesity is a multicomponent phenomenon that involves not only nasal epithelial receptor activation, but also different brain areas, such as the limbic system, thalamus and piriform cortex, as well as amygdala, which project to the orbitofrontal cortex [88].

Beside the afore-mentioned factors that bring about a decrease in ONS, we should not forget to mention the role of cytokine-induced (generalized or tongue-specific) inflammation in obesity. The mouse taste bud cells have been shown to produce both TNF-α and IL-10 in the microenvironment of taste papillae [89,90]. In a plausible study, Kaufman et al. [74] showed that an increase in TNF-α in the tongue of obese mice was associated with a significant reduction in taste bud and taste progenitor cells in tongue papillae. Moreover, TNF-α^null^ mice were protected from obesity-induced reduced number of taste bud cells, and administration of exogenous TNF-α brought back taste buds to degeneration [91]. The adipose-specific deletion of Sel1L in mice maintained on a high-fat diet resulted in reduced adiposity and showed neither an increase in TNF-α concentrations nor any sign of taste bud cell atrophy. These observations clearly indicate that TNF-α released from hyperplasic/inflamed adipose tissue in obesity may trigger a loss in gustatory taste perception. Moreover, LPS-induced inflammation was also found to decrease the lifespan of mature taste bud cells [92].

As regards olfactory perception, inflammation and obesity, a link between apoptosis and inflammation has been recently reported in the olfactory mucosa of obese mice fed with a moderate high-fat diet, where a significant increase in activated caspase-3 was associated with a marked loss of olfactory sensory neurons and their axonal projections, paralleled with an increased expression of Iba-1, suggesting an increase in proinflammatory cells [92]. Hence, if the diet-induced obese mice are re-fed a normal diet and return to normal weight, the loss in olfactory perception is also reinstated. In vitro, TNF-α has been shown to induce cell death in olfactory epithelial explants [93]. In transgenic mice, the expression of TNF-α resulted in the loss of olfactory neurons and odor perception. As regards IL-6, its concentrations were found elevated in the blood of patients suffering from hyposmia [94,95]. A perusal of above-mentions observations clearly suggests that obesity is associated with the loss of ONS, and inflammation in the oro-naso epithelia plays an important role in this phenomenon.

## 5. Conclusions

Figure 2 shows that SARS-CoV-2 infection will install (or aggravate) an inflammatory state both in the lingual and nasal epithelia. In the lingual taste buds, the virus-induced inflammation will attenuate the gustatory perception of different taste qualities, whereas in the olfactory sensory neurons, the virus-triggered inflammation may contribute to decreased olfactory perception of odorants. It is also possible that SARS-CoV-2, by penetrating the olfactory bulb, may enter the brainstem and modulate ONS. Why do all COVID-19 patients not exhibit a change in ONS perception? It is possible that the alteration in oro-olfactory epithelium functions might be secondary to viral infection, which may depend on genetic (or epigenetic) and other life-style-related build-up of the patients. Nonetheless, we can infer that obese subjects are at high risk for SARS-CoV-2 infection as they already exhibit a low ONS capacity for different taste modalities. Hence, the existing gustatory and olfactory sensory deficiency, due to obesity, will mask SARS-CoV-2-induced diminished taste and smell sensation and, thus, may aggravate the patient’s health. SARS-CoV-2 infection may further aggravate the ONS functions; mask the obesity-induced inflammation, including loss of taste and smell; and render the obese subjects more vulnerable and prone to severe pathophysiological consequences such as RTI, leading to death.

## 6. Perspectives

By now, we have observational/self-reported studies, but data regarding the duration and the time of the onset and reversal of ONS symptoms in this infection are lacking. We need a complete follow-up study of these patients as a function of time on the loss of ONS. As mentioned previously, we also lack the proper set-up for the detection of olfactory and taste thresholds. We still do not know whether SARS-CoV-2 infection alters the taste bud renewal/turn-over and taste bud physiology either upstream or downstream of the detection of sapid molecules. It is too early to predict clearly that SARS-CoV-2-induced changes in ONS might be due to its direct or indirect deleterious effects on brain regions such as the insula, caudal orbitofrontal and anterior cingulate cortex that control the integration of both taste and smell information [96]. While we have mentioned that tongue epithelium expresses ACE2 receptors [54], we still do not know which cell type (type I, II or III) expresses this receptor. This information will be important to correlate the loss of a particular taste modality as type II cells express sweet, bitter and umami receptors; type I cells express salt receptors; and type III cells are involved in sour sensing [74,75].

The vistas in the eating behavioral physiology with regard to SARS-CoV-2 infection require more detailed investigations in COVID-19 patients as gustatory and olfactory receptors are also expressed in other tissues such as those in the gut, which is the main site of the release of small peptides (such as cholecystokinin and peptide-YY) that control eating behavior via the vagus nerve [97]. Similarly, the olfactory bulb also expresses receptors for a number of appetite-regulating hormones and peptides such as insulin, leptin, ghrelin and orexin [98]. It is now well established that the gut microbiome of obese subjects is shifted from Bacteriodetes to Fermecutes, a pro-inflammatory phylum, and the effects of this change on SARS-CoV-2 infection susceptibility should be explored in the future. Does this viral infection promote a particular microbiome in the gut and ONS epithelia? A recent report has outlined that there is a significant persistent alteration in the gut microbiome in COVID-19 patients [99]. Can the strategies to alter the intestinal microbiota decrease the severity of SARS-CoV-2 infection? We think that SARS-CoV-2 infection is much more dangerous than what is reported now and a significant amount of clinical information remains undiscovered.
